# Gata3 is required in late proneurosensory development for proper sensory cell formation and organization

**DOI:** 10.1038/s41598-023-39707-0

**Published:** 2023-08-03

**Authors:** Paige V. Blinkiewicz, Makayla R. Long, Zachary A. Stoner, Elizabeth M. Ketchum, Sydney N. Sheltz-Kempf, Jeremy S. Duncan

**Affiliations:** 1 Department of Biological Sciences, Western Michigan University, Kalamazoo, MI USA; 2 Department of Biomedical Sciences, Western Michigan School of Medicine, Kalamazoo, MI USA; 3 Present Address: Section On Sensory Cell Regeneration and Development, National Institute on Deafness and Other Communication Disorders, National Institutes of Health, Bethesda, MD 20892 USA; 4 Present Address: Department of Neurology, University of Minnesota, Minneapolis, MN USA

**Keywords:** Cell biology, Developmental biology, Neuroscience

## Abstract

It has previously been shown that the zinc-finger transcription factor *Gata3* has dynamic expression within the inner ear throughout embryonic development and is essential for cochlear neurosensory development. However, the temporal window for which *Gata3* is required for proper formation of the cochlear neurosensory epithelia remains unclear. To investigate the role of *Gata3* in cochlear neurosensory development in the late prosensory stages, we used the *Sox2-cre*^*ERT2*^ mouse line to target and conditionally delete *Gata3* at E11.5, a timepoint before cells have fully committed to a neurosensory fate. While the inner ears of *Sox2-cre*^*ERT2*^*: Gata3 f/f* mice appear normal with no gross structural defects, the sensory cells in the organ of Corti are partially lost and disorganized in an increasing severity from base to apex. Additionally, spiral ganglion neurons display aberrant peripheral projections, including increased distances between radial bundles and disorganization upon reaching the organ of Corti. Furthermore, heterozygous *Sox2-cre*^*ERT2*^*: Gata3 f/*+ mice show a reduced aberrant phenotype in comparison to the homozygous mutant, supporting the hypothesis that *Gata3* is not only required for proper formation at the later proneurosensory stage, but also that a specific expression level of *Gata3* is required. Therefore, this study provides evidence that *Gata3* plays a time-sensitive and dose-dependent role in the development of sensory and neuronal cells in late proneurosensory stages.

## Introduction

The mammalian inner ear is comprised of six unique sensory organs that are responsible for our senses of hearing and balance. The cochlea contains the organ of Corti (OC), the hearing organ, which is comprised of mechanosensory hair cells (HCs) and their corresponding supporting cells (SCs). HCs transduce sound energy into electrical impulses via innervation by spiral ganglion neurons (SGNs), which then project into the hindbrain for further auditory processing. The early development of these three cell types has been extensively studied, but there are still gaps in knowledge regarding the transcriptional regulatory networks that control the spatial and temporal aspects of their development at later proneurosensory stages.

The inner ear is derived from the otic placode, which invaginates to form the otic cup before developing into the otocyst around embryonic day 8 (E8)^1,2^. While several transcription factors are important for neurosensory development in this time frame, the zinc-finger transcription factor *Gata3* is particularly interesting due to its dynamic expression throughout inner ear development. While *Gata3* is initially expressed as early as E8.5 throughout the otocyst, by E10.5 its expression is restricted to the proneurosensory regions^3–9^. *Gata3* continues to be expressed in SGNs until postnatal day 14 (P14) and remains highly expressed in SCs, with lower levels of expression in HCs, throughout adulthood^10–14^. Therefore, it has been postulated that *Gata3* plays an important and dynamic role in inner ear development and neurosensory cell formation during this temporal window.

Previous studies have shown that loss of *Gata3* in the early proneurosensory region around E8.5 leads to loss of all cochlear neurosensory cells^5,15^, while loss of *Gata3* 1 day later around E9.5 leads to a patchy loss of HCs and SCs, and disorganization and patchy loss of SGNs^6^. Other studies have investigated the role of *Gata3* postnatally in the maintenance of HCs and SCs^11,12^. These studies found that *Gata3* is necessary later on to maintain outer hair cells (OHCs) and to functionally develop inner hair cells (IHCs), while loss of *Gata3* from postnatal SCs results in an increase in some types of SCs through downregulation of other genes. However, there exists a gap in knowledge about the role of *Gata3* later in embryonic development during the period where proneurosensory cells begin differentiating into HCs, SCs, and SGNs. Specifically, it remains to be seen how long *Gata3* is required for proper embryonic development of neurosensory cells before switching to its postnatal maintenance role. Additionally, while we know the presence of *Gata3* is necessary for proper neurosensory development, it is also critical that expression levels of *Gata3* are precise for maintenance and function of neurosensory cells. For example, both *Gata3* haploinsufficiency and *Gata3* over-expression (as a result of gene duplication) cause human hypoparathyroidism, sensorineural deafness, and renal dysplasia (HDR) syndrome^16–21^. While the triad of symptoms of HDR syndrome range in severity, nearly all patients exhibit deafness^16,19,22,23^. Uniquely, deafness is the only symptom of HDR syndrome which can present singularly^16,19,21,23^. This suggests that not only is continued expression of *Gata3* required for proper inner ear development, but specific levels of *Gata3* are also required. Continued investigation of the dose-dependent requirements of *Gata3* will also contribute to the field’s overall understanding of inner ear gene regulatory networks.

In this study, we explored the window of developmental plasticity which is governed by *Gata3* as a follow-up to previous studies showing that loss of *Gata3* is detrimental to cochlear neurosensory epithelia^5–7,10,21,24,25^. Using the *Sox2-cre*^*ERT2*^ mouse line^26^, we conditionally deleted *Gata3* from proneurosensory cells via tamoxifen injection at E11.5. Our results show that deletion of *Gata3* causes severe loss and disorganization of HCs, SCs, and SGNs in a basal to apical gradient, with a more severe phenotype presenting in the apex. Interestingly, the mutant ears were morphologically normal in that they presented a full-length cochlea structure, unlike previous *Gata3* deletion studies^5,6^. Overall, we show that while *Gata3* is not necessary at E11.5 for overall morphological development and elongation of the cochlea structure, *Gata3* is required in later proneurosensory development for proper neuronal and cochlear sensory epithelia cell formation.

## Results

### *Gata3* is deleted from HCs, SCs, and SGNs at E11.5

**Figure 1:**
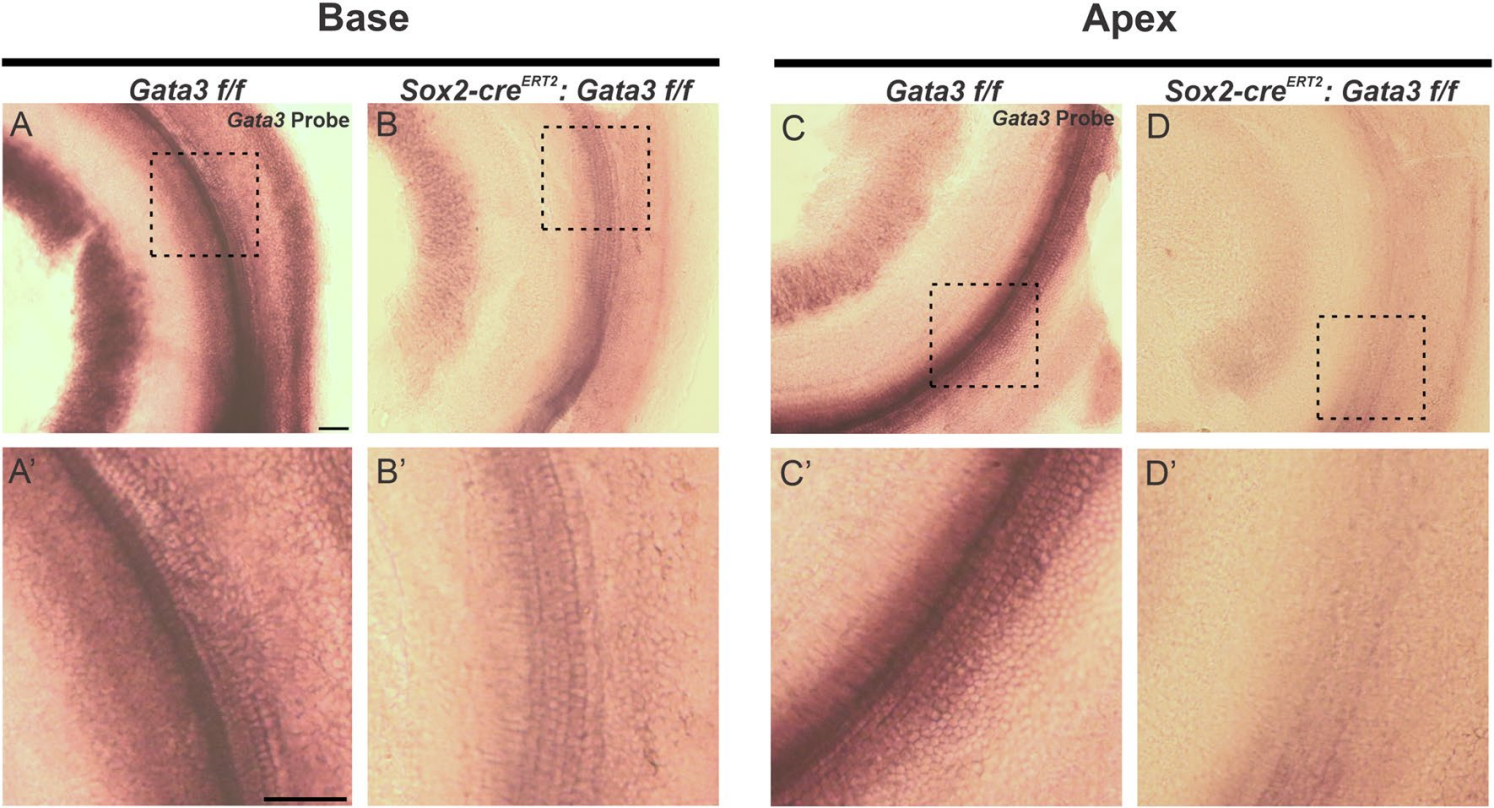
*Gata3* is conditionally deleted from HCs, SCs, and SGNs at E11.5 (**A**–**D′**) Whole mount in situ hybridization was performed with a *Gata3* riboprobe on a *Gata3 f/f* control and a *Sox2-cre*^*ERT2*^*: Gata3 f/f* mutant and imaged at the cochlear base and apex. *Gata3* expression appears in the HCs, SCs, and SGNs of the control and is absent in the HCs and SCs and decreased in the SGNs of the homozygote mutant. Scale bar: 100 µm.

Previous studies have characterized *Sox2-cre*^*ERT2*^ expression at the placode stage (E8.5), otocyst stage (E10.5), and the late otocyst stage (E12.5)^27–32^. At E10.5, *Sox2* is present in both the nonsensory cochlear floor and roof^31^. At E11.5, *Sox2* is expressed in the anteroventral region of the otocyst^30^. By E12.5, *Sox2* is expressed in OC sensory cells and greater epithelial ridge (GER) cells^29,31^. In order to confirm knockout of *Gata3* from HCs, SCs, and SGNs, in situ hybridization was performed using a *Gata3* riboprobe. While the control showed high expression of *Gata3* in all cell types from base to apex, the homozygous mutant displayed minimal expression in the HCs and SCs and greatly reduced expression in the SGN cell bodies (Fig. 1A–D′), demonstrating that our model reduces levels of *Gata3* in the cell types of interest.

### *Gata3* is required for sustained formation and organization of HCs

**Figure 2:**
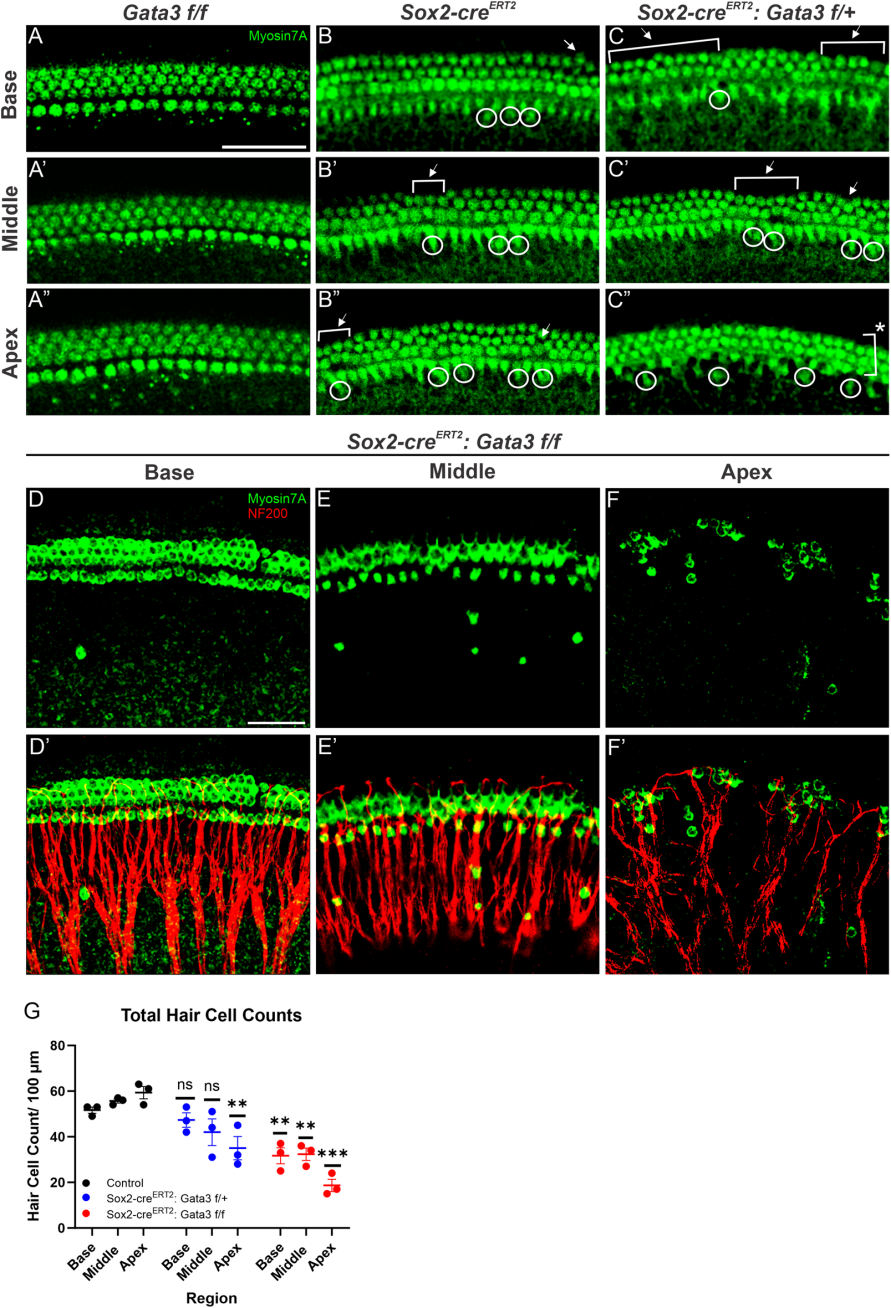
Deletion of *Gata3* results in loss of HCs in a basal to apical gradient (**A**–**F′**) Representative images from the basal, middle, and apical regions of the cochlea for HCs indicated by MYOSIN7A^+^ staining. Two different controls were used, *Gata3 f/f* and *Sox2-cre*^*ERT2*^, in order to account for the haploinsufficent phenotype of the Cre line used. Both the heterozygous and homozygous mutant show IHC duplets (white circles) and missing rows of OHCs (white brackets), while the homozygous mutant also shows ectopic HCs in the GER. (**G**) Total hair cell quantification was performed in the base, middle, and apex in 100 µm sections using a One-way ANOVA with post hoc Dunnett’s test (P** ≤ 0.01; P*** ≤ 0.001) Scale bar: 50 µm.

Previous *Gata3* conditional knockout (CKO) models show either no HC development or only patches of HCs^5–7,10^. We therefore wanted to assess the effect of deleting *Gata3* at E11.5 on HC development. For this assessment, two different controls were used: *Gata3 f/f* (Fig. 2A–A″) and *Sox2-cre*^*ERT2*^ (Fig. 2B–B″). Other studies have demonstrated that the knock-in *Sox2-cre*^*ERT2*^ line shows IHC duplets, which was confirmed in our study (Fig. 2B–B″; white circles). It was important to investigate the IHC duplets in the *Sox2-cre*^*ERT2*^*: Gata3 f/*+ (heterozygous) mutant compared to the *Sox2-cre*^*ERT2*^ control to ensure that any resultant phenotype in the heterozygous mutant was not attributed to using this Cre line (Fig. 2C–C″). While the base, middle, and apex of the heterozygous mutant all contained IHC duplets similar to the *Sox2-cre*^*ERT2*^ control, it should be noted that the third row of OHCs was lost in the middle and into the apical region of the OC (Fig. 2C–C″). The heterozygous phenotype showed continuous formation of HCs from base to apex; however, conditional deletion of both alleles of *Gata3* (homozygous mutants) resulted in disruptions in HC formation in the apical region, similar to the previous *Gata3* CKO study that observed HC patches in the absence of *Gata3*^6^. *Sox2-cre*^*ERT2*^*: Gata3 f/f* mutants also showed a worsening phenotype compared to the heterozygous mutants. In homozygous mutants, the base contained all three rows of OHCs, but a progressive loss of OHC rows occurs spatially along the OC (Fig. 2D–D′). Only two rows of OHCs were present in the middle region and almost no rows of OHCs were present in the apex (Fig. 2E–F″). Quantification of the total number of HCs in the basal, middle, and apical regions was performed between *Gata3 f/f* controls (base: 51.67 ± 2.31; middle: 55.67 ± 1.53; apex: 59.33 ± 4.73), *Sox2-cre*^*ERT2*^*: Gata3 f/*+, and *Sox2-cre*^*ERT2*^*: Gata3 f/f* ears, as indicated by mean ± standard deviation of three ears, in 100 µm sections, with significance set at P = 0.05 using a one-way ANOVA (Fig. 2G). In heterozygous mutants, loss of HCs was only statistically significant in the apex (35.00 ± 8.89, P = 0.0063); HC loss in the base and middle were not significantly different from control (47.33 ± 5.51, P = 0.4956; 42.00 ± 10.15, P = 0.0735, respectively). However, the homozygous mutant revealed a statistically significant loss of HCs from all three regions of the cochlea (base: 31. 67 ± 6.11, P = 0.0046; middle: 32.33 ± 4.73; P = 0.0083, apex: 18.67 ± 4.73, P = 0.0005, respectively).

Homozygous mutants contained MYOSIN7A^+^ cells in the GER along the length of the cochlea, with the highest number appearing in the apex, similar to a postnatal *Gata3* CKO from SCs using this same Cre line^11^. Ectopic HCs have previously been seen in the GER in both CKO and over-expressor models^33–38^. While ectopic HCs are generally not seen in combination with missing rows of OHCs, previous studies have shown that loss of *Gata3* results in missing OHCs postnatally^12,21^. The phenotype of both ectopic HCs and missing rows of OHCs as a result of embryonic loss of *Gata3* is unique and further supports a role for *Gata3* in this specific temporal window in HCs.

### *Gata3* is required for corresponding SC formation and organization

**Figure 3:**
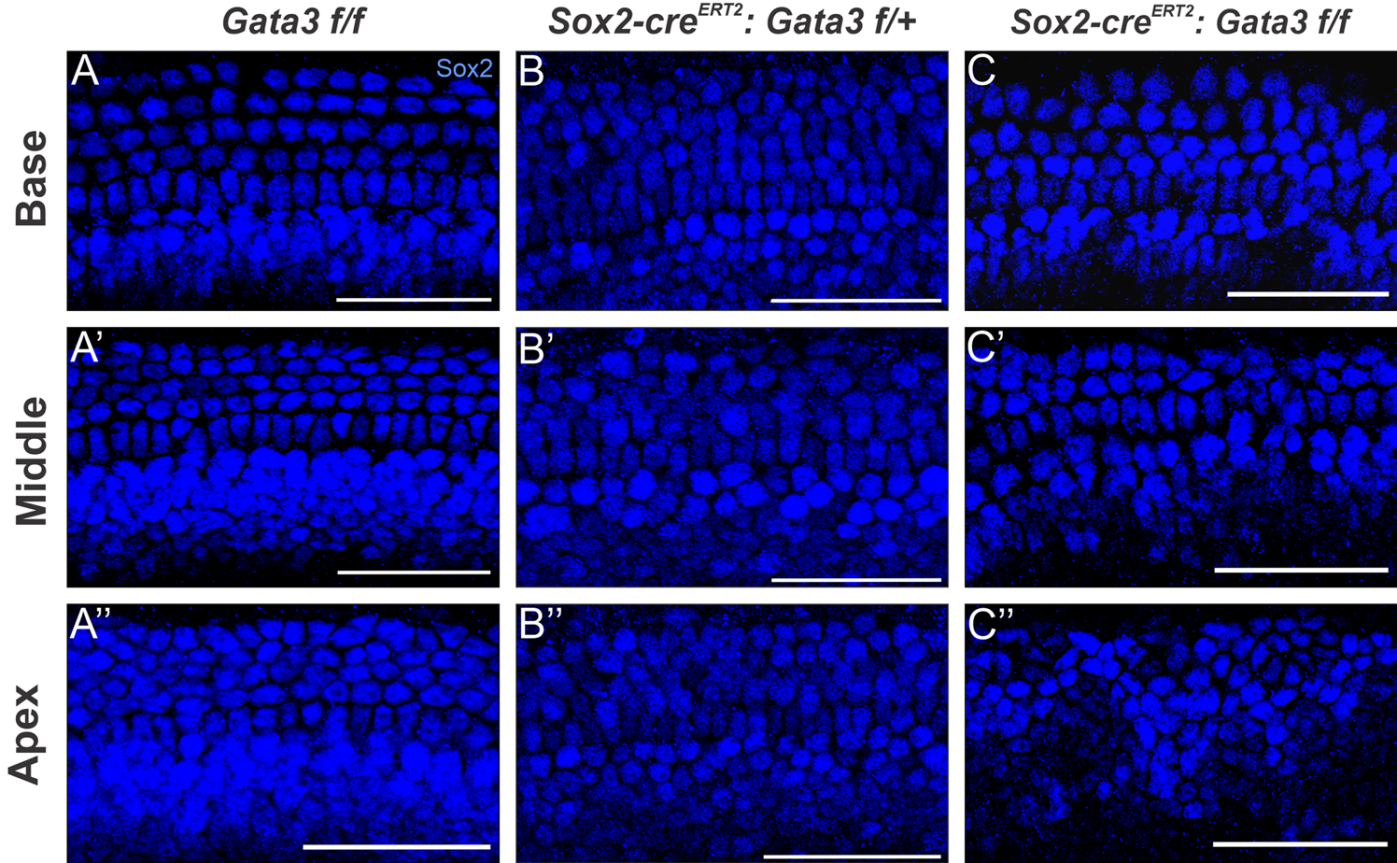
Deletion of *Gata3* results in loss of SCs in a basal to apical gradient (**A**–**C″**) Representative images from the basal, middle, and apical regions of the cochlea showing SCs indicated by SOX2^+^ staining. The homozygous mutant shows a worsening disorganization of SCs from base to apex, with entire rows of SCs missing in the middle and apex. Scale bar: 25 µm.

Previous studies that have examined the effects of deleting *Gata3* on inner ear development have shown either no SC development or disorganization and limited formation of SCs in variable phenotypic severity^5,6,10^. However, SCs were present in our model throughout the majority of the cochlear length, as indicated by SOX2^+^ SCs (Fig. 3). Similar to the HC phenotype in this model, heterozygous mutants showed continuous formation of SCs with some loss and disorganization of SCs in the apex (Fig. 3B–B″). As with HCs, homozygous mutants showed a worsening phenotype compared to heterozygous mutants (Fig. 3C–C″). The basal and middle regions contained disorganized SCs and complete loss of some outer SC rows in the middle region. The apex contained the most severe phenotype in which SCs appear to cluster together, similar to the SC phenotype seen in other *Gata3* CKO studies^10^. Ultimately, the phenotype of HCs and SCs in homozygous mutants are consistent in their progressive loss from base to apex. While SC disorganization in our model is similar to the phenotype seen in another *Gata3* CKO study, it is important to note that this previous study did not observe ectopic HCs in the GER^10^. Therefore, further experiments are needed in order to tease apart the specific requirement for *Gata3* within proneurosensory development and differentiation to determine if *Gata3* deletion in one cell population can influence another cell population.

### *Gata3* is required for organization of SGN peripheral projections

**Figure 4:**
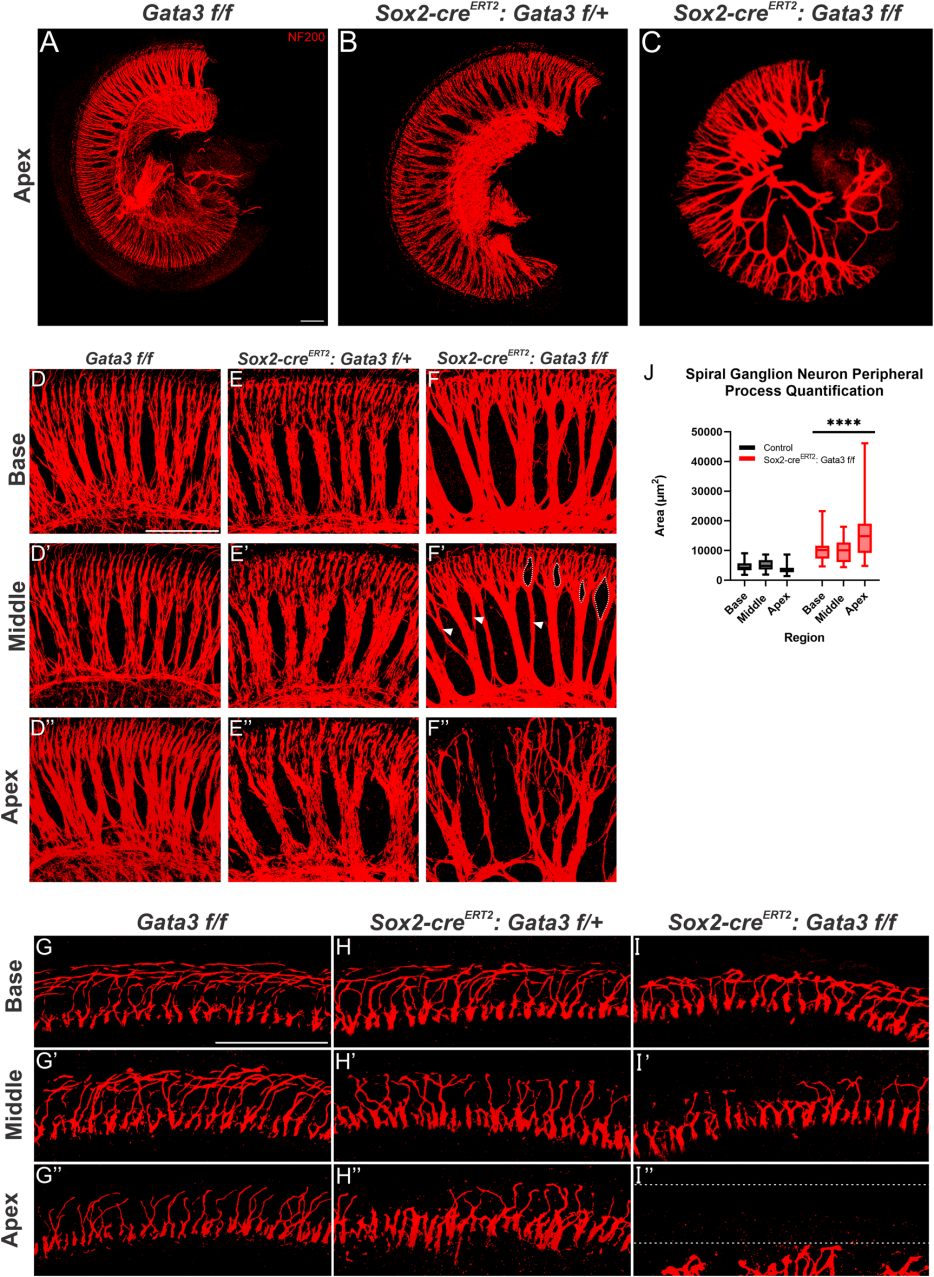
Deletion of *Gata3* results in fewer SGN processes in a worsening gradient from base to apex (**A**–**C**) Overview of SGN processes in the control, heterozygous mutant, and homozygous mutant apex labeled by NF200 staining. (**D**,**D′**,**D″**) Radial bundles of a control sample. (**E**,**E′**,**E″**) Radial bundles of a heterozygous mutant. Slightly increased space between the radial bundles is observed in all three regions. (**F**–**F″**) Radial bundles of a homozygous mutant. The distances between radial bundles are significantly increased relative to those of the control sample. The dotted white outline and white arrowheads in F’ indicate increased branching in the middle. The radial bundles in (**F″**) exhibit an even greater degree of branching as well as an increase and irregular distance between the fibers. (**F″**) (**G**,**G′**,**G″**) Peripheral projections of the control where they reach the OC are well organized in the control. (**H**) In the heterozygous mutant, peripheral neurites are present and relatively organized. (**H′**) Peripheral neurites in the middle of the heterozygous mutant have some peripheral projections that misturn towards the apex instead of the base. Additionally, there appear to be fewer neurites present than in the control. (**H″**) In the apex of the heterozygous mutant, there are fewer peripheral projections and those that are present show disorganization relative to the control sample. (**I**) In the base of the homozygous mutant, peripheral projections are present but are fewer in number and show an increased misturning of neurites towards the apex. (**I′**) The middle of the homozygous mutant has drastically fewer neurites reaching the OC relative to the control, particularly those neurites that should project to the OHC region. (**I″**) The apex of the homozygous mutant has some peripheral neurites approaching the IHC region of the OC but no peripheral neurites extending to the OHC region. The dotted white lines indicate where the OC should be. (**J**) Quantification of the distances between radial bundles in all three regions of control, heterozygous mutant, and homozygous mutant samples. The distance between radial bundles is greater in homozygote mutant samples than in controls and homozygous samples show greater variability in the distance between radial bundles as indicated by a TTEST (P**** ≤ 0.0001). Scale bar: 100 µm.

Previous studies examining the effect of *Gata3* deletion from the proneurosensory region of the developing otocyst noted a severe reduction in the number of SGNs present in CKO mutant samples and SGNs that did form had aberrant projection patterns towards the developing OC^6,10^. A study in which *Gata3* deletion was restricted to SGNs resulted in present SGNs; however, peripheral projections were disorganized^14,24^. As the *Sox2-cre*^*ERT2*^ model results in *Gata3* deletion from multiple cell types, including SGNs, we hypothesized that deletion of *Gata3* from multiple cell types would result in an aberrant neuronal phenotype. We first examined peripheral projections in a *Sox2-cre*^*ERT2*^ mutant sample to determine whether the Cre knock-in displays a SGN phenotype. When compared with control samples (Fig. 4D–D″), there was no obvious difference in SGN number or organization (data not shown), suggesting that any potential phenotype in mutant samples would be attributed to deletion of *Gata3*. We then examined the peripheral projections in a heterozygous mutant. The gross organization of SGNs in the heterozygous mutant is disrupted when compared to control samples in the base and middle regions (Fig. 4E–E′). However, the radial bundles in the apex of the heterozygous mutant appear to have an increased area separating them relative to the control (4F”). The homozygous mutants had an even more striking phenotype, including a further increase in the distances between radial bundles relative to the control and progressively increased disorganization along the length of the cochlea (Fig. 4F–F″). The mutant base, middle, and apex (Fig. 4F–F″) reveal irregular distances between radial bundles, which were statistically significant (P < 0.0001; Fig. 4J), in addition to extra branches from radial bundles towards the OC. The method for radial bundle area quantification can be found as Supplementary Fig. S1 online. This phenotype was most profound in the apex (Fig. 4F″). Additionally, the areas between radial bundles in mutant samples were highly variable, further supporting that loss of *Gata3* results in disorganization of peripheral projections of SGNs (Fig. 4J).

We then examined the peripheral projections where the neurites reach the OC (Fig. 4G–I″). The basal region of the heterozygous mutant was comparable to the control (Fig. 4G,H), but peripheral projections were progressively fewer and became disorganized, with increasing severity from the middle to apical regions (Fig. 4G′–G″,H′–H″). Upon examination of homozygous mutants, peripheral projections in the base appeared slightly disorganized upon reaching the OC. Additionally, the density of neurites in the homozygous mutant appeared to be less when compared to the base of the control (Fig. 4I). The disorganization of the neurites and decreased density was even more pronounced in the middle and apical regions of the homozygous mutant (Fig. 4I′–I″). Fewer neurites projected into the OHC region of the OC in the middle and few-to-no neurites projected to the OHC region in the apex. In these regions, not all neurites that were present within the OHC region properly turned towards the base, but rather, turned towards the apex.

Based upon our results, *Gata3* expression is important for the formation of radial bundles with regards to appropriate density and distance between bundles, as well as for proper branching patterns and overall organization. Additionally, *Gata3* is needed for peripheral neurites to reach the OC, particularly into the OHC region. Importantly, the loss of *Gata3* has a phenotype that progressively worsens along the length of the cochlea, with the most severe phenotype observed in the apex.

### *Gata3* is required for proper central pathfinding of SGNs

**Figure 5:**
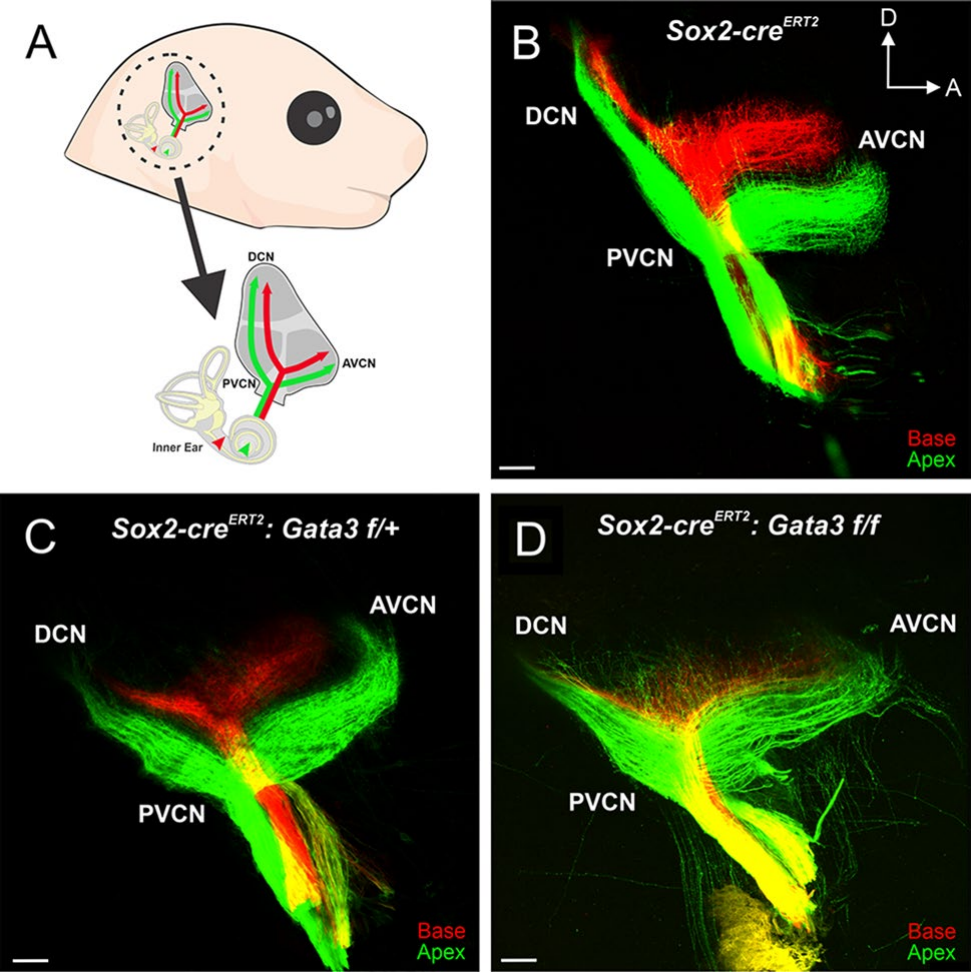
SGN central pathfinding is altered by *Gata3* deletion (**A**) Schematic view of lipophilic dye placement and visualization of SGNs in the CN. (**B**–**D**) Lipophilic dye was applied to the base (red) and apex (green) of control and mutant cochleas at E18.5 and their central projections were analyzed. (**B**) In the *Sox2-cre*^*ERT2*^ control, SGNs bifurcate and send processes towards the AVCN and DCN/PVCN. Basal and apical SGN fibers also remain segregated throughout the CN (**C**) Heterozygous SGNs bifurcate and maintain tonotopic segregation similar to controls. (**D**) Homozygous mutants have aberrant SGN central projections with apical fibers projecting more dorsally and sometimes projecting outside the CN. Additionally, some SGN neurites project outside cranial nerve VIII before reaching the CN.

Given that homozygous mutants display aberrant peripheral projections of SGNs, with the phenotype progressively increasing in severity from base to apex (Fig. 4), we next investigated whether central projections of SGNs to the cochlear nucleus (CN) were also affected. Previous studies examining the role of *Gata3* in SGN central pathfinding have shown varied results depending on the location and timing of *Gata3* deletion^6,24^. Early deletion of *Gata3* throughout the entire inner ear at E9.5 results in central SGN fibers bifurcating at several branch points with terminal fibers projecting non-specifically throughout the CN^6^. However, deletion of *Gata3* exclusively from delaminated SGNs at E9.5 results in normal projection of SGNs to the CN with tonotopy maintained^24^. Taken together these two studies suggest that *Gata3* may affect SGN central pathfinding in a cell non-autonomous and time-dependent manner. In order to investigate this further, lipophilic dyes were applied to the base (red) and apex (green) of *Sox2-cre*^*ERT2*^ control, heterozygous mutant, and homozygous mutant cochleas (Fig. 5A) to visualize the projections of SGNs into the CN. *Sox2-cre*^*ERT2*^ control SGNs entered the hindbrain and bifurcated, sending ascending and descending process towards the anteroventral cochlear nucleus (AVCN) and dorsal cochlear nucleus (DCN)/posteroventral cochlear nucleus (PVCN), respectively (Fig. 5B). *Sox2-cre*^*ERT2*^ control SGNs remained segregated, with basal fibers extending more dorsally and apical fibers more ventrally (Fig. 5B). This stereotyped central wiring was also maintained in heterozygous samples (Fig. 5C). In contrast, SGNs in homozygous mice displayed less segregation between apical and basal fibers. Apical fibers often projected more dorsally into spaces occupied by basal fibers. Additionally, some apical fibers, upon reaching the hindbrain, projected outside of cranial nerve VIII into areas outside of the CN (Fig. 5D). These results provide further evidence that *Gata3* plays an important role in the development and wiring of SGNs centrally. Our data, along with previous studies^6,24^, suggest that *Gata3* is acting in a cell non-autonomous manner at or before E11.5 to promote proper central wiring of SGNs. Further investigations are needed to elucidate what cell populations require early *Gata3* expression in order to promote proper central pathfinding of SGNs.

### *Gata3* deletion at E11.5 results in full morphologic development of the cochlear duct and vestibular system, but shows progressive neurosensory epithelial loss and disorganization

**Figure 6:**
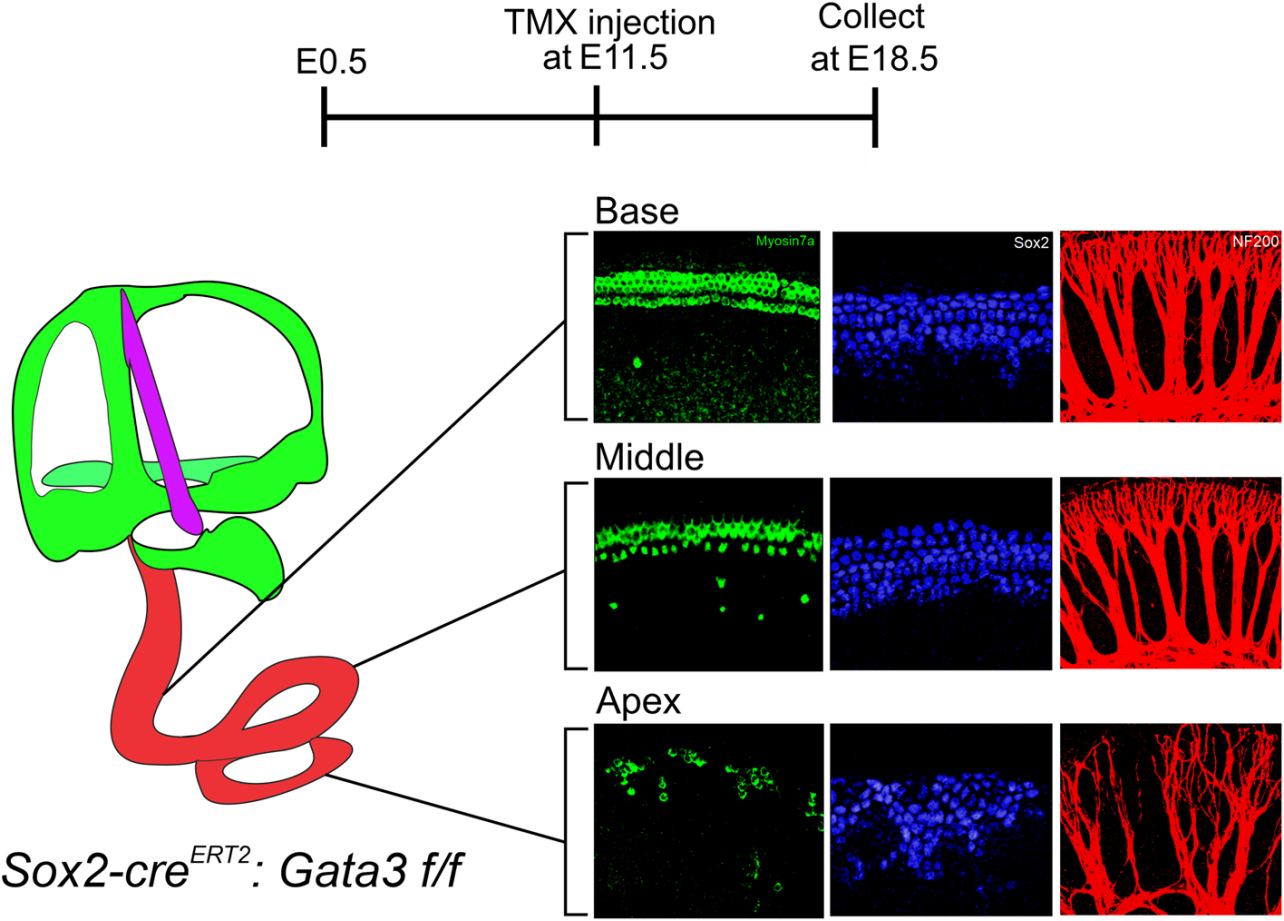
Loss of *Gata3* is depicted by the timeline with TMX injection at E11.5 and collection of tissue at E18.5. A *Gata3* mutant ear at E18.5, indicates the cochlea (red), vestibular system (green), and endolymphatic duct (purple). Images are representative of the phenotype observed along the length of the cochlea and are taken from Figs. 2, 3, and 4. Our study deleted *Gata3* at E11.5, later than previous studies, and found that the overall morphology of the inner ear is intact, unlike in the previous studies. In addition, there was a loss of sensory cells with increasing severity from base to apex, indicating that *Gata3* is still required for their formation and organization.

Previous *Gata3* deletion studies have shown a variety of phenotypes that include cochlear structure and cochlear neurosensory epithelia cell defects^5–7,10,21,24,25^. *Gata3* null mice display a severely truncated cochlea and vestibular system devoid of sensory epithelia except for a small patch of HCs and SGNs in a portion of the saccule^5,7^. *Gata3* deletion at E8.5 using the *Foxg1-cre* mouse line resulted in a truncated cochlea, which contained no HCs and abnormal morphologic development of the vestibular system^6^. *Gata3* deletion at E9.5 using the *Pax2-cre* mouse line resulted in similar morphologic defects, including a truncated cochlea and abnormal vestibular system. However, unlike deletion at E8.5, deletion at E9.5 resulted in patchy sensory cell development and a limited population of SGNs^6,10^. In studies where *Gata3* has been conditionally deleted from only SGNs, HCs and SCs form properly^24,25^. We contribute results for *Gata3* deletion at E11.5, a time in development in which proneurosensory cell differentiation is occurring. Our findings show that *Gata3* deletion at E11.5 results in a morphologically sound structure with a full-length cochlea and well-developed vestibular system (data not shown). Within the homozygous mutant, the sensory cells in the OC are mostly present and have a varying phenotype depending on the cochlear region. In the homozygous mutant cochlear base, HCs and SCs are present with only mild disorganization (Fig. 6), while the homozygous mutant basal radial bundles have larger spacing than normal but the neurons are relatively organized. This contrasts the phenotype seen in the apex where the peripheral projection density of the mutant apex is decreased and those projections which are present appear disorganized (Fig. 6). Additionally, the tonotopy of SGN central projections is largely maintained within the CN in both heterozygous and homozygous mutants (Fig. 5). In comparison, the mutant apical HCs are severely reduced to patchy clusters with some ectopic HCs that appear in the GER, while the apical SCs are not organized in rows and instead cluster together (Fig. 6). Our data demonstrates a role for *Gata3* in all neurosensory cells after their initial specification.

## Discussion

*Gata3* was previously shown to be necessary for both proper cochlear structure and cochlear neurosensory epithelia cell formation early in inner ear development when its expression is high throughout the entire otocyst^5–7,10,21,39^. However, the role of *Gata3* in HC, SC, and SGN formation after its restriction to the proneurosensory region was unknown. Our study reveals novel findings that *Gata3* plays both a necessary and dose-dependent role in the formation and organization of neurosensory cell types but does not have an impact on the overall morphology of the inner ear at this specific developmental time point.

This project contributes new knowledge about the role of *Gata3* in proneurosensory epithelia cell formation in a temporal window that fills a gap between previous studies investigating *Gata3* deletion. Our results demonstrate that deletion of *Gata3* from the proneurosensory domain at E11.5 results in a fully-formed cochlear duct (data not shown), regardless of the single or dual loss of *Gata3* alleles. Therefore, *Gata3* is not required for morphologic development at or after E11.5. Given that previous *Gata3* deletion studies did not see normal morphology of the cochlea^5,6^, it is intriguing that deletion of *Gata3* approximately 2 days later than previous studies results in a morphologically sound inner ear with a fully formed cochlea structure. We hypothesize that the resulting phenotype from this deletion of *Gata3* prior to neurosensory cell differentiation may be the result of abnormal differentiation that occurs as a result of the loss of a key transcription factor, rather than cell cycle exit.

Previous lineage tracing analysis using the *Sox2-cre*^*ERT2*^ line has shown that tamoxifen administration at E11.5 results in nearly complete labeling of neurosensory cells in the apex, compared to sparser labeling in the base^29^. Consistent with this, our model appears to show increased recombination efficiency of *Gata3* in the apex when compared to the base (Fig. 1). Despite this, homozygous mutant cochleas still displays near-complete deletion of *Gata3* in the base when compared to controls (Fig. 1). Deletion of *Gata3* at E11.5 using the *Sox2-cre*^*ERT2*^ line results in formation of HCs, SCs and SGNs. However, they are highly disorganized and this phenotype increases in severity from base to apex. In *Gata3* heterozygous null mice, OHC loss occurs in the absence of *Gata3*^12,21,40^. This phenotype is mirrored in our study, despite the difference in timing at which *Gata3* is deleted. It is also noteworthy that the heterozygous mutant had a subtler phenotype compared to the homozygous mutant, suggesting that precise levels of *Gata3* are needed for proper formation and organization of the proneurosensory epithelia. If precise levels of *Gata3* are truly necessary, then increased levels of *Gata3* should also have a phenotype in our model. Several other over-expressor studies have been published that demonstrate ectopic HCs in the GER^33–36,38^. Previous studies have even used the *Gata3* over-expressor model in combination with upregulation of other sensory genes in order to increase the efficiency of ectopic HC formation^35,36^. Investigating the over-expression of *Gata3* using this *Sox2-cre*^*ERT2*^ mouse line would be useful in determining any detrimental effects of elevated levels of *Gata3* in the cochlea. While this would further elucidate the specific role of *Gata3* in the cochlea, the investigation of *Gata3* over-expression is especially pertinent since extra alleles of *Gata3* have also been known to cause HDR syndrome^20^.

Finally, it should also be noted that *Gata3* was deleted from three different cell types: HCs, SCs, and SGNs. While these cell types work together, it is unclear if loss of *Gata3* in just one of the cell types is enhancing the overall phenotype we see in our model. Despite that HCs in our model appear to be innervated by SGNs throughout the entire OC, we are unable to determine if the HC disorganization is causing the improper SGN peripheral projections when using the *Sox2-cre*^*ERT2*^ model, or vice versa. Likewise, since the SCs and HCs are connected via tight junctions in the OC, our model is unable to determine if a phenotype in one of these cell types is exacerbating the overall phenotype. Therefore, in order to tease apart the role of *Gata3* during proneurosensory development, future studies could use cell-specific Cre lines to delete *Gata3*. Comparison of the phenotype in our model to *Gata3* CKO in HC-specific, SC-specific, or SGN-specific lines could help elucidate the exact role of *Gata3* in this stage of proneurosensory development.

In conclusion, our work demonstrates that *Gata3* is essential for proper cochlear neurosensory epithelia cell development and organization in the late proneurosensory stage at E11.5. Because our study demonstrates a phenotype in the heterozygous mutant in addition to a more severe phenotype in the homozygous mutant, we propose that correct levels of *Gata3* are also required for proper development. Furthermore, our study performs the latest embryonic *Gata3* deletion from the inner ear yet. We contribute to the understanding of neurosensory development that *Gata3* is required for proper formation and organization of cochlear afferent neurons and sensory epithelia at E11.5, but not for overall cochlear morphology.

## Methods

### Mouse model and genotyping

All animal care and procedures were approved by Western Michigan University Institutional Animal Care and Use Committee (IACUC) following the guidelines for use of laboratory animals (IACUC #20-11-01). All experiments were carried out in accordance with the ARRIVE guidelines, and all methods were carried out in compliance with all relevant regulations. The *Sox2-cre*^*ERT2*^ (Jackson Labs)^26^ and *Gata3 Flox* (provided by Dr. Maxime Bouchard^41^) mouse strains were used in this study. *Sox2-cre*^*ERT2*^ males were bred with *Gata3 f/f* females to produce males that were *Sox2-cre*^*ERT2*^*: Gata3 f/*+, who were viable. *Sox2-cre*^*ERT2*^*: Gata3 f/f* mice were produced by breeding *Sox2-cre*^*ERT2*^*: Gata3 f/*+ or *Sox2-cre*^*ERT2*^*: Gata3 f/f* males with *Gata3 f/f* or *Gata3 f/*+ females. Genotyping was performed using the following primers: Cre 5′ CCT GTT TTG CAC GTT CAC CG 3′ and 5′ ATG CTT CTG TCC GTT TGC CG 3′ yield a 280 base pair (bp) mutant, IL2 5′ CTA GGC CAC AGA ATT GAA AGA TCT 3′ and 5′ GTA GGT GGA AAT TCT AGC ATC ATC C 3′ yield a 324 bp control band, and *Gata3* 5′ GAT TCA GTC TCC CTC CTT CTT C 3′ yield a 430 bp mutant band and 5′ GTT CAC ACA CTC CCT GCC TTC TG 3′ yield a 400 bp control band. Breedings were performed with E0.5 specified as noon on the day of vaginal plug. Pregnant females received an intraperitoneal injection of 3 mg/40 g tamoxifen and 2 mg/40 g progesterone at E11.5 between 9 and 11 a.m.^30^. On the day of collection, the pregnant female was given a lethal intraperitoneal injection of Avertin (500 mg/kg 2.2.2-tribromoethanol). Embryos were dissected from the uterus, perfused with 4% paraformaldehyde (PFA) and stored at 4 °C. All images are representative of at least three biological replicates.

### Whole-mount immunohistochemistry

Whole mount immunohistochemistry was performed on previously fixed tissue^42^. Ears were washed in phosphate buffered saline (PBS), then washed five times five minutes in PBS/0.05% Tween20 followed by blocking for one hour in 5% normal donkey serum, 1% bovine serum albumin, and 0.5% TritonX-100 in PBS. The tissue was incubated in primary antibodies, diluted in blocking buffer, at 4 °C for three nights. The following primary antibodies were used: MYO6 Rabbit (Sigma; 1:1000), MYOSIN7A Mouse (DSHB; 1:200), MYSOIN7A Rabbit (Proteus Biosciences, Inc.; 1:500), Neurofilament 200 (NF200) HC Chicken (Aves; 1:200), and SOX2 Rabbit (Sigma; 1:500). Next, the tissue was washed four times thirty minutes, followed by overnight incubation at 4 °C in secondary antibody in blocking buffer. Secondary antibodies were conjugated to Alexa flour anti-Mouse 488, anti-Rabbit 488, anti-Chicken 555, anti-Goat 647, or anti-Rabbit 647 (Life Tech; 1:1000). Nuclei were labeled using Hoescht Dye (1:2000), received as a gift from Bernd Fritzsch. Images were taken on either a Nikon C2 confocal microscope or a Leica Stellaris 5 confocal microscope and images were compiled in ImageJ and edited in CorelPhoto Paint (Version 19.0; 2017).

### Total hair cell quantification

For total hair cell quantification, shown in Fig. 2, whole-mount immunohistochemistry samples that were stained with MYO6/MYOSIN7A/MYOSINVIIA were quantified in the base, middle, and apical regions in 100 µm sections using FIJI imaging software (Version 1.8.0_172). Hair cell counts were recorded in GraphPad Prism (Version 8.0.0) and a One-Way ANOVA and post hoc Dunnett’s test were performed with significance set at P < 0.05. All counts are representative of three biological replicates.

### Spiral ganglion neuron peripheral process quantification

For radial bundle quantification, shown in Fig. 4 and Supplementary Fig. S1 online, cochleas were imaged at the same magnification in the base, middle, and apex for control and homozygote samples. Using FIJI imaging software (Version 1.8.0_66), eight spaces between radial bundles in each region were outlined and measured. All area results were recorded in GraphPad Prism (Version 9.1.2) and a TTEST analysis was performed. Data point plot graphs were constructed, and significance was set at P < 0.05.

### In situ hybridization

*Gata3* mRNA labeling was achieved using a previously described in situ hybridization protocol^42^. Mice were fixed in 4% PFA and inner ears were dissected in 0.4% PFA. Control ears and experimental ears were run together throughout the experiment to ensure both ears received the same experimental conditions. Ears were dehydrated overnight in 100% methanol and rehydrated through a graded methanol series. Ears were digested with Proteinase K in PBS (Ambion, Austin, TX, USA). Samples were hybridized overnight at 60 °C to the *Gata3* riboprobe in hybridization solution consisting of 50% (v/v) formamide, 50% 2X saline sodium citrate (SSC), and 6% (w/v) dextran sulphate. Unbound probe was removed by performing washes with 2X SSC. Samples were then incubated with anti-digoxigenin antibody conjugated with alkaline phosphatase (Roche Diagnostics GmbH, Mannheim, Germany) overnight at room temperature. Ears were extensively washed with 1X washing buffer throughout the day, then left overnight in 1X washing buffer at room temperature. Samples were then incubated at room temperature in detection buffer (Roche) before being thoroughly saturated with nitroblue phosphate/5-bromo, 4-chloro, 3-indolil phosphate (BM purple substrate, Roche). Control and mutant samples were developed in BM purple for the same length of time. Ears were mounted in glycerol on a slide and imaged with a Nikon Eclipse E600 microscope and Canon EOS Rebel T7i camera. Images were edited in CorelDraw (version 19.0; 2017).

### Lipophilic dye tracing

Neuronal tracing of spiral ganglion neurons was conducted as previously described^42^. Briefly, the lateral half of the inner ear was exposed, and pieces of lipophilic dye-soaked paper was inserted into the base (NeuroVue^®^ Red) and apex (NeuroVue^®^ Maroon) of the cochlea. Heads were then placed into glass vials filled with 4% PFA and incubated at 37 °C for 3 days to allow for proper dye diffusion. Following incubation, the brains were removed, and the brain stem was flat mounted with the lateral side facing up in glycerol on a slide and imaged within 1 h of dissection. All imaging was performed using a Leica Stellaris 5 confocal microscope with LAS X software and images were compiled in ImageJ and edited in CorelPhoto Paint (Version 19.0; 2017).

### Supplementary Information

Supplementary Figure S1.

## Data Availability

Data is freely available upon request. Requests for data should be addressed to ZAS (email: zach.stoner@nih.gov).
