# Supplementary material for: Gata3 is required in late proneurosensory development for proper sensory cell formation and organization
Source: Sci Rep. 2023 Aug 3;13:12573. doi: 10.1038/s41598-023-39707-0 (PMC10400699; doi:10.1038/s41598-023-39707-0)
Supplement: Supplementary file 1 — Supplementary Figure S1. [file 41598_2023_39707_MOESM1_ESM.pdf]

Gata3 is Required in Late Proneurosensory Development for Proper Sensory Cell Formation and Organization

Paige V. Blinkiewicz[1,+], Makayla R. Long[1,+], Zachary A. Stoner[1,3,*], Elizabeth M. Ketchum[1], Sydney N. Sheltz-Kempf[1], and Jeremy S. Duncan[1,2,4,*]

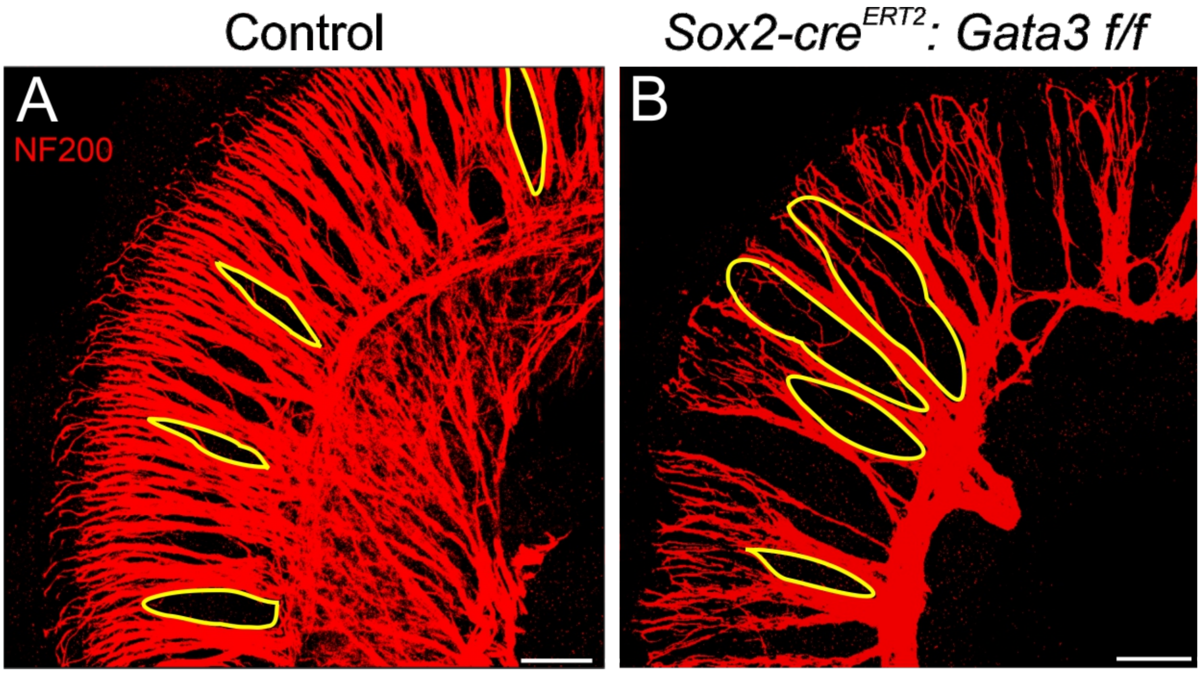

Supplementary Figure S1. Distances between radial bundles were quantified in the apical regions of control and mutant cochleas. The yellow regions above represent outlines drawn to define the areas between the bundles. FIJI imaging software was then used to calculate these areas. **(A)** shows a sample quantification from the apex of a control cochlea and (**B)** shows a sample from the apex of a *Sox2-cre*^{ERT2}*: Gata3 f/f* cochlea. Scale bar: 100 µm
